# Impact of MSWI Bottom Ash Codisposed with MSW on Landfill Stabilization with Different Operational Modes

**DOI:** 10.1155/2014/167197

**Published:** 2014-03-23

**Authors:** Wen-Bing Li, Jun Yao, Zaffar Malik, Gen-Di Zhou, Ming Dong, Dong-Sheng Shen

**Affiliations:** ^1^Key Laboratory of Hangzhou City for Ecosystem Protection and Restoration, College of Life and Environmental Science, Hangzhou Normal University, Hangzhou 310036, China; ^2^College of Environmental and Resource Sciences, Zhejiang University, Hangzhou 310058, China; ^3^College of Life Science, Taizhou University, Linhai 317000, China; ^4^Zhejiang Provincial Key Laboratory of Solid Waste Treatment and Recycling, Zhejiang Gongshang University, Hangzhou 310018, China

## Abstract

The aim of the study was to investigate the impact of municipal solid waste incinerator (MSWI) bottom ash (BA) codisposed with municipal solid waste (MSW) on landfill stabilization according to the leachate quality in terms of organic matter and nitrogen contents. Six simulated landfills, that is, three conventional and three recirculated, were employed with different ratios of MSWI BA to MSW. The results depicted that, after 275-day operation, the ratio of MSWI BA to fresh refuse of 1 : 10 (V : V) in the landfill was still not enough to provide sufficient acid-neutralizing capacity for a high organic matter composition of MSW over 45.5% (w/w), while the ratio of MSWI BA to fresh refuse of 1 : 5 (V : V) could act on it. Among the six experimental landfills, leachate quality only was improved in the landfill operated with the BA addition (the ratio of MSWI BA to fresh refuse of 1 : 5 (V : V)) and leachate recirculation.

## 1. Introduction

During the past three decades, an unprecedented increase in the amount of solid waste was concomitant with the tremendously developing economy in China. No other country in the world has ever experienced such a fast and large increase in solid waste quantities that is occurring in China now [1]. Landfill is predominant in the disposal of MSW, due to the advantage of cost effectiveness and the large accommodation of waste in amount and types. For instance, in 2006, the United States generated 251 million tons of MSW, about 67% of which were disposed in landfills [2]. In Greece, the main destination for MSW is landfills [3]. In China, about 190 million tons of MSW were produced annually, nearly 90% of which were disposed by landfills [1]. However, with the increase in landfill costs, scarcity of landfill sites, and enhancement of public environmental consciousness, the government of China has been urged to consider alternative disposal methods. Incineration, due to its primary advantages of hygienic control, volume and mass reduction, and energy recovery, has become an attractive method of MSW disposal [4, 5]. During an incineration process, various solid residues, such as BA, fly ash (FA), and air pollution control residues, are produced. BA, including grate siftings, is the main waste stream, accounting for approximately 80% of total solid residues [6]. Nowadays in China, MSWI BA is either reused as a secondary construction material, such as for coffering road and making brick, or used as daily cover material for landfill [7, 8]. In 2008, the Ministry of Environmental Protection of China announced that MSWI BA was allowed to be disposed in landfills directly by adopting “Standard for pollution control on the landfill site of municipal solid waste” (GB 16889-2008, China). Therefore, the amount of MWSI BA disposed in landfills will increase in China.

Recently, several experimental studies reported the feasibility of codisposal MSWI BA with MSW in landfills. Banks and Lo [7] assessed the effect of MSWI BA on the biodegradation of organic materials and found that the addition of BA had beneficial effects on the degradation process of landfilled refuse, based on the variation of pH, total organic carbon of leachate, and landfill gas production. Lo [9] investigated the behavior of heavy metals and the alkali metals and their potential effects on anaerobic codigestion and concluded that BA as a soil cover might have beneficial effects on landfill practice, such as the increase in gas production and landfill settlement. Lo and Liao [10] also investigated the potential metal-releasing and acid-neutralizing capacity (ANC) of MSWI BA and FA in landfill sites and reported that MSWI BA and FA had beneficial rather than detrimental effects on landfill stabilization. Boni et al. [11] studied the effect of different disposal (mixed or layered) and management strategies (anaerobic or semiaerobic conditions) on landfills, which are codisposal with pretreated waste (organic fraction of MSW (OFMSW)) and BA, and showed that aerobic management and layered configuration could lead to more rapid biological and mechanical stabilization of the bulk waste than mixed BA and OFMSW in anaerobic conditions. Lo et al. [12] investigated the effects of MSWI FA and BA on the anaerobic codigestion of OFMSW with FA or BA and showed that the addition of ashes could improve the MSW anaerobic digestion and enhance the biogas production rates.

Although MSWI BA contains high level of alkali, heavy, and trace metals, its impact on the degradation process of landfilled refuse codisposed with MSWI BA and MSW is still not clearly known. In addition, the results reported above cannot provide enough valuable reference for the MSW treatment in China. First of all, most of the experiments reported above used mimic waste or the pretreated MSW, and the proportion of food and fruit waste was lower than that in China (usually higher than 45%). Thus, both the volatile fatty acid (VFA) and organic matter concentration in leachate during landfill stabilization, especially in the acid phase, are higher than these studies above. Secondly, the mechanical-biological and/or thermal pretreatment of MSW needs costly technological equipment. In addition, these pretreatment techniques were usually used for the MSW of high calorific value. However, the relatively high water content of waste (74%), a common characteristic of the refuse produced in Asian countries, will lead to low calorific values [13]. Therefore, in view of these two reasons, the mechanical-biological and/or thermal pretreatment techniques of MSW are not suitable for Asian countries, such as China. Although the simulated waste used by Lo et al. [12] could represent a similar organic matter proportion of municipal refuse, they established landfill anaerobic conditions by the addition of sludge from a wastewater treatment plant. However, in terms of leachate volume and the cost of treatment, most of landfills do not allow the addition of sludge due to high water contents of sludge. Therefore, in order to understand the effect of codisposal of MSWI BA with MSW on landfill stabilization with high contents of food and fruit waste, the fundamental information of codisposal of MSWI BA and MSW needs to be obtained. Unfortunately, to our knowledge, no such study has been conducted.

The aim of the study was to investigate the effects of codisposal of MSWI BA and MSW on the stabilization of the simulated landfill by monitoring leachate quality including organic matter and nitrogen contents. The influence of the additional ratio of MSWI BA to MSW on the degradation of the landfilled refuse was also discussed. These results will provide a better understanding of the feasibility of codisposal of MSWI BA with MSW in the landfill in the developing countries.

## 2. Materials and Methods

### 2.1. Experimental Set-Up

#### 2.1.1. Simulated Landfill Design and Operation

**Figure 1:**
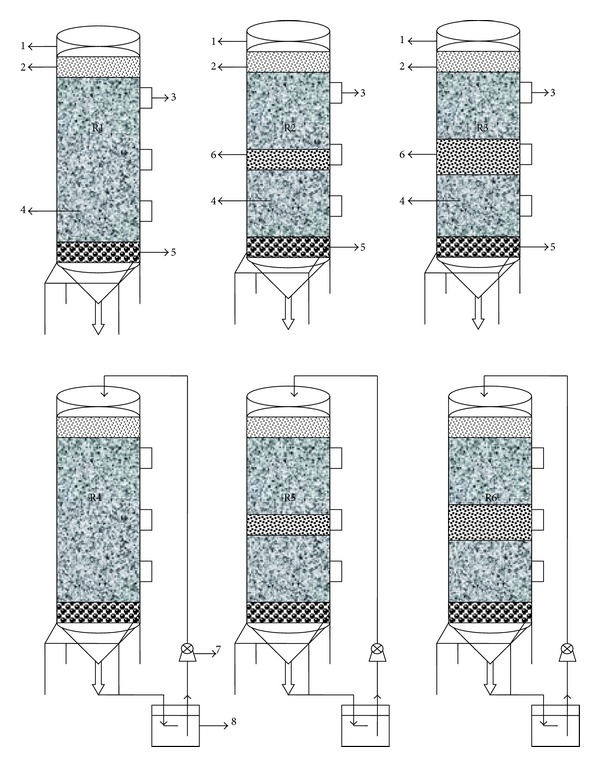
Schematic of the six simulated landfills in the experiment: (1) headspace, (2) sandy layer, (3) sampling port, (4) municipal solid waste (MSW), (5) gravel layer, (6) municipal solid waste incinerator (MSWI) residue, (7) peristaltic, and (8) leachate collection tank.

In the experiment, six simulated landfills were employed, coded as R1, R2, R3, R4, R5, and R6. The schematic configurations of the experimental setup are shown in Figure 1. R1, R2, and R3 were conventional landfills (CL) where leachate leached, while R4, R5, and R6 were recirculated landfills (RL) from which the leachate was collected and directly recycled by a peristaltic pump. R1 and R4 were only loaded with fresh refuse and served as controls for the other four simulated landfills with different ratios of MSWI BA to fresh refuse by layered configuration. The ratio of MSWI BA to fresh refuse was 1 : 10 (V : V) in the MSWI-added simulated landfills of R2 and R5, while it was 1 : 5 (V : V) in R3 and R6.

The simulated landfill, with an internal diameter of 320 mm and a height of 1050 mm, was constructed using polypropylene with a thickness of 10 mm. Five ports (a diameter of 50 mm) were designed in each simulated landfill, of which the two inlet/outlet ports at the top lid were used for gas emission and leachate recycling (only for the types of RL), the two ports at the side of simulated landfill were used for sampling refuse, and the remaining one at the bottom of simulated landfill was used for leachate drainage. A gravel layer of 50 mm height was put at the bottom before loading refuse, and a sand layer of 50 mm height was placed on the top of landfilled refuse in each simulated landfill to provide even distribution of leachate and to prevent clogging of the leachate outlets. According to the initial water content and weight of the refuse in different simulated landfills, water was added to obtain the initial moisture content of landfilled refuse of 75% (w/w) in the simulated landfills, which is reported to be an initial rapid decomposition threshold for the anaerobic organic refuse mineralization in bioreactor landfills [14, 15]. After loading refuse, all the simulated landfills were sealed with a gasket and silicone sealant and then operated at room temperature. Leachate was collected and stored in leachate collection tanks. Leachate of CL was discarded without further treatment, while leachate of RL was continuously recirculated using pumps with adjusted flow rates varying with leachate volume every day, except for the first week when no recycled leachate was fed to the simulated landfill. The recycled leachate volume was equal to the effluent leachate volume each day.

#### 2.1.2. Characteristics of MSW and MSWI

The fresh refuse was collected from Kaixuan transport station of Hangzhou (Zhejiang, East China). After being transported to the lab, the refuse was shredded to less than 10–30 mm. The refuse was thoroughly mixed, and then loaded into landfills at a wet density of 680 kg m^−3^. Moisture content of refuse was 54%. The composition of experimental refuse was as follows (by wet weight, w/w): food and fruit waste (such as pineapple and citrus sinensis), 45.5%; dust, 5.2%; papers, 9.5%; plastics, 8.5%; wood, 0.7%; cellulose textile, 0.2%; brick, 5.8%; residue, 24.6%.

Fresh MSWI BA sample was taken from Green Energy MSWI plant in Zhejiang province, East China. The plant consisted of three parallel stoker incinerators with a MSW treatment capacity of 650 t d^−1^. The MSW without any industrial solid waste for the incinerators was collected from several residential areas in Hangzhou. The operating temperature of the incinerators was 850–1100°C, and the residence time of waste in the incinerator was about 50 min. BA had been treated by water quenching and magnetic separation before being sampled. The sampling period lasted for 5 days. Approximately 25 kg of fresh BA sample was taken daily from the plant and a total of 125 kg BA sample was obtained. Then, the BA sample was mingled and homogenized. About 25 kg of the MSWI BA was oven-dried and grounded into less than 154**μ**m with a grinder (Retsch BB51, Germany) for bulk composition analysis. The remaining part was used for the simulated landfill experiment.

The contents of individual elements in the fresh BA sample were analyzed after the sample was digested as described previously [5]. In brief, about 0.5 g of air-dried sample was added into a Teflon beaker. The sample was added with 2.5 mL HNO_3_ and 2.5 mL HClO_4_ and then heated at 150°C for 2-3 h. After cooling, the digested product was added with 2.5 mL HClO_4_ and 5 mL HF and heated at 150°C for 15 min, and then the residue was added with another 5 mL HF and heated, again, until the liquid became dried. The residue was dissolved using 5 mL HNO_3_ and then diluted to 100 mL. The element concentrations in the solution were determined by Inductively Coupled Plasma Optical Emission Spectrometer (ICP-OES) (Thermo Electron Corporation IRIS/AP, USA).

### 2.2. Acid-Neutralizing Capacity Experiment

ANC experiment was conducted by the batch titration procedure suggested by Johnson et al. [16]. Each 2.5 g of MSWI bottom ash sample was placed in 25 previously acid washed polyethylene bottles and thoroughly rinsed with deionized water. Acidic solutions (250 mL) were produced from degassed deionized water and 1.0 M of HNO_3_ and were added to the samples ranging from 0 to 4.8 mmol H^+^· g^−1^ MSWI bottom ash. The solutions were continually flushed with N_2_ to avoid contact with the atmosphere and shaken for 24 h at 25°C. The solution pH values were determined immediately.

### 2.3. Sampling and Analytical Procedures

Leachate samples were collected weekly from leachate outlet ports (~100 mL). The same volume of water (~100 mL water) was added into the leachate to balance the volume of leachate from RL before recirculation. Leachate samples were collected at the bottom of the simulated landfill. Physical-chemical characteristics of leachate, such as pH, chemical oxygen demand (COD_Cr_), dissolved organic carbon (DOC), volatile fatty acid (VFA), total nitrogen (TN), ammonium nitrogen (NH_4_^+^-N), nitrate nitrogen (NO_3_^−^-N), and nitrite nitrogen (NO_2_^−^-N) were measured mainly by the Standard Methods of the State Environmental Protection Administration of PR China. COD_Cr_ was measured using the dichromate method (GB 11914-89, China). DOC, after filtration through a 0.45**μ**m filter, was determined by total organic carbon analyser (SHIMADZU TOC-V CPH, Japan). VFA was measured by acidified ethylene glycol colorimetric method [17]. TN was measured by alkaline potassium persulfate digestion-UV spectrophotometric method (GB 11894-89, China), and NH_4_^+^-N was measured by Nessler's reagent colorimetric method (GB 7479-87, China). In addition, the pH values were measured by a pHS-digital pH meter (DELTA 320). For the analyses of metal concentration, the leachate sample was predigested with concentrated HNO_3_ and HCl (1 : 3) according to the standard method [18]. The rest of the items were detected by standard methods adopted for the examination of water and wastewater [18]. All the analyses were performed in triplicate.

## 3. Results and Discussion

### 3.1. Acid-Neutralizing Capacity of MSWI Bottom Ash

**Figure 2:**
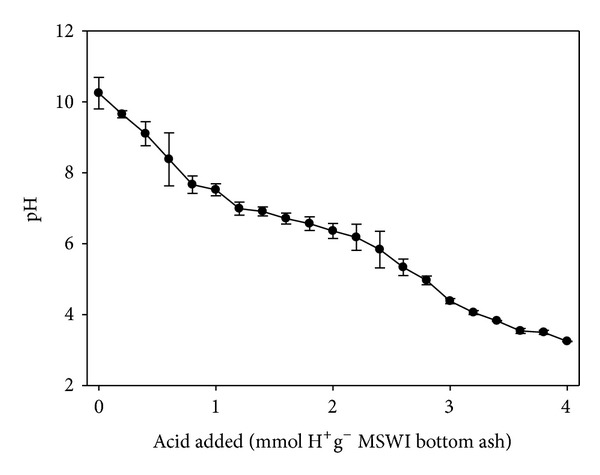
The pH titration curve of municipal solid waste incinerator (MSWI) bottom ash.

ANC is usually a measure for the overall buffering capacity against acidification for MSWI bottom ash. As was shown in Figure 2, the initial pH was 10.3 without addition of acid to the solution and then decreased gradually with the addition of acid to the solution. According to the acids titration curve, ANC_pH=7.5_ of around 1 mequiv·g^−1^ of bottom ash was obtained. When 4.0 mmol H^+^·g^−1^ MSWI bottom ash was added, the pH decreased to 3.3, the lowest in this study. Therefore, the MSWI bottom ash used in the present experiment has the potential capacity to neutralize the part of the volatile fatty acids derived from the leachate of simulated landfill.

### 3.2. Characteristics of Leachate VFA and pH

**Figure 3:**
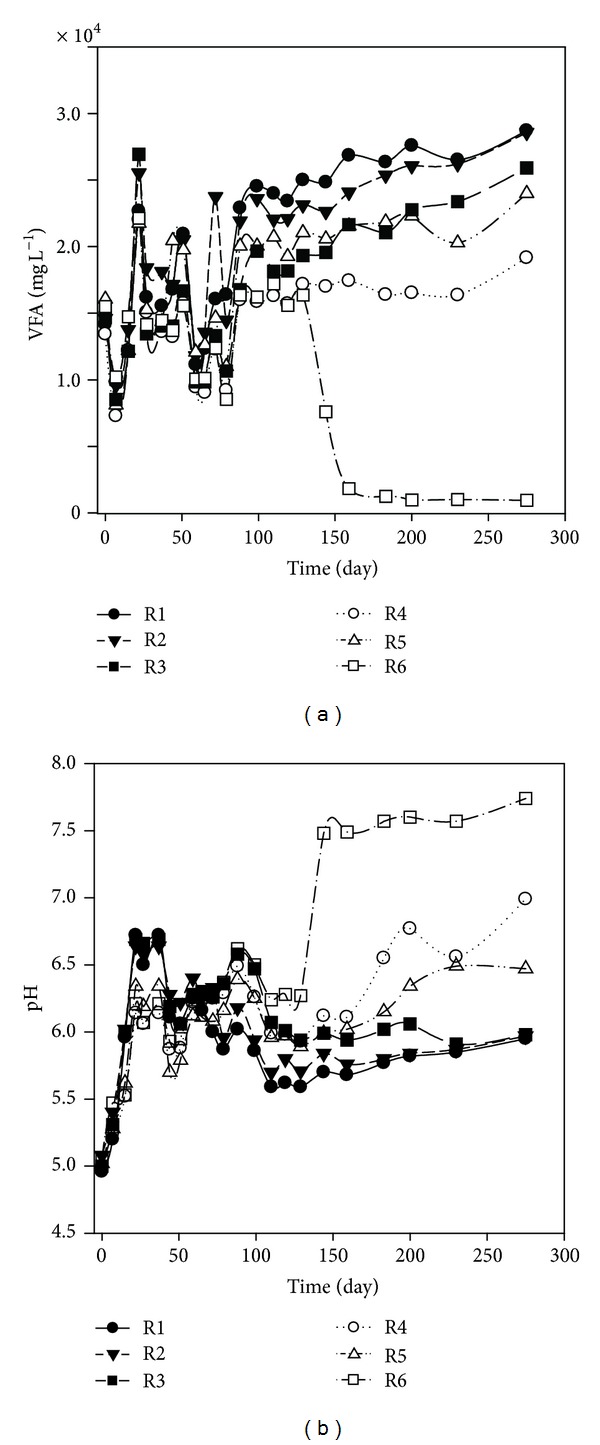
Time evolutions of VFA (a) and pH (b) in the leachate of the simulated landfill during operation.

**Figure 4:**
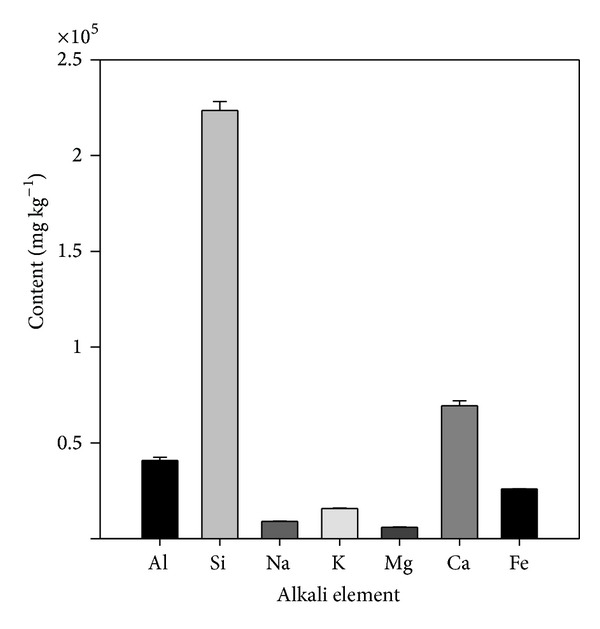
Bulk chemical composition of the MSWI bottom ash sample.

One of the most important intermediates in the anaerobic digestion process is VFA, which has a good relationship with pH value. Therefore, VFA has been used as a process performance indicator of anaerobic reactors [19]. As can be seen from Figure 3(a), the VFA concentration presented similar trends in the leachate from the six simulated landfills at the beginning of 89 days. All the leachate VFA concentrations of the six simulated landfills decreased at the first week and then increased linearly and reached the maximum values of 22000 mg L^−1^ to 26900 mg L^−1^. The rapid increase in VFA in the six simulated landfills was attributed to the accumulation of soluble long-chain fatty acids in the leachate. Most of the soluble organic refuse was converted into VFA in a short time due to the rapid multiplication of acidogens, a bacterial group with a minimum doubling time of around 30 minutes. As a result, the leachate VFA concentration reached peak value within 21 days. Afterwards, all the leachate VFA concentrations for the six simulated landfills decreased and kept within the range of 13000 to 16000 mg L^−1^ except a small fluctuation on day 51. From then on, the VFA concentrations presented different trends. The leachate VFA concentrations of R1, R2, R3, R4, and R5 increased and finally were maintained approximately at 28700 mg L^−1^, 28500 mg L^−1^, 26000 mg L^−1^, 19000 mg L^−1^, and 24000 mg L^−1^, respectively. No significant change in the leachate VFA concentration of R6, within the range of 16400 mg L^−1^ to 17200 mg L^−1^, was found from day 89 to day 129. However, the leachate VFA concentration of R6 decreased sharply from 16400 mg L^−1^ to 1250 mg L^−1^ and then was maintained at about 1000 mg L^−1^, which was one order of magnitude lower than the corresponding values in other five simulated landfills. As can be seen from Figure 4, the alkali metal contents of the MSWI BA, such as Al, Fe, Ca, Mg, K, and Na, which were thought to be the sources of alkalinity providing the acids neutralizing capacity to the landfills, were 9040 ± 178 mg kg^−1^ to 69400 ± 2610 mg kg^−1^. In the present study, the proportion of food and fruit waste was as high as 45.5%. In addition, the residue (24.6%) was almost comprised of organic matter. Therefore, high organic composition of refuse leads to high concentration of VFA in leachate, which can only be neutralized by enough alkali content. According to the results, we hypothesized that the ratio of MSWI BA to fresh refuse 1 : 5 (V : V) in the landfill was enough to provide sufficient acids neutralizing capacity for high organic compositions of MSW (higher than 45.5%).

All leachate pH values were in accordance with the concentration of VFA in the six simulated landfills. The “ensiling” problems were observed in the three simulated conventional landfills. As was shown in Figure 3(b), all the leachate pH values were 5.00 and increased gradually during the first month. The low pH values might mainly result from the alpha hydroxyl acid released by the degradation of pineapple and other fruit wastes, which were the main constituent of the food and fruit waste in our study. Afterwards, no significant change was found in the leachate pH of the three simulated conventional landfills, but the leachate pH increased with the increasing ratio of the BA addition. The leachate pH values of R1, R2, and R3 kept within the range of 5.62–6.11, 5.70–6.33, and 5.93–6.58. Low pH values observed during the whole process in the three simulated landfills may be ascribed to the production of low alkalinity in these reactors, which is not enough for maintaining the neutral pH and buffering the producing VFA [20, 21]. Although R2 and R3 were loaded with MSWI BA with different proportions, less amounts of alkali metals contained in the BA without leachate recirculation were found compared to the simulated landfill with leachate recirculation. Leachate recirculation not only can increase the moisture content of landfilled refuse, but also provides good conditions for the release of the nutrition/nutrients and alkali metals from MSWI BA. Therefore, no significant difference was found among the three simulated landfills, namely, R1, R2, and R3. The leachate pH values of the three simulated recirculated landfills (R4, R5, and R6) were all higher than CL, especially for R6 (the ratio of MSWI BA to fresh refuse was 1 : 5 (V : V)), and the leachate pH value of R6 increased linearly from day 119 to day 144 and finally kept stable at 7.57–7.74. The sudden increase in pH value in simulated landfill R6 on day 119 might result from the hydrolyzing and fermentation of VFA to carbon dioxide and methane, which agrees with the decrease in leachate VFA concentrations. These results indicated that the coeffect of BA addition and leachate recirculation was beneficial to solve the ensiling problems and favored a faster degraded and more stable state compared without leachate recirculation and/or BA addition.

### 3.3. Characteristics of Leachate COD_Cr_ and DOC

**Figure 5:**
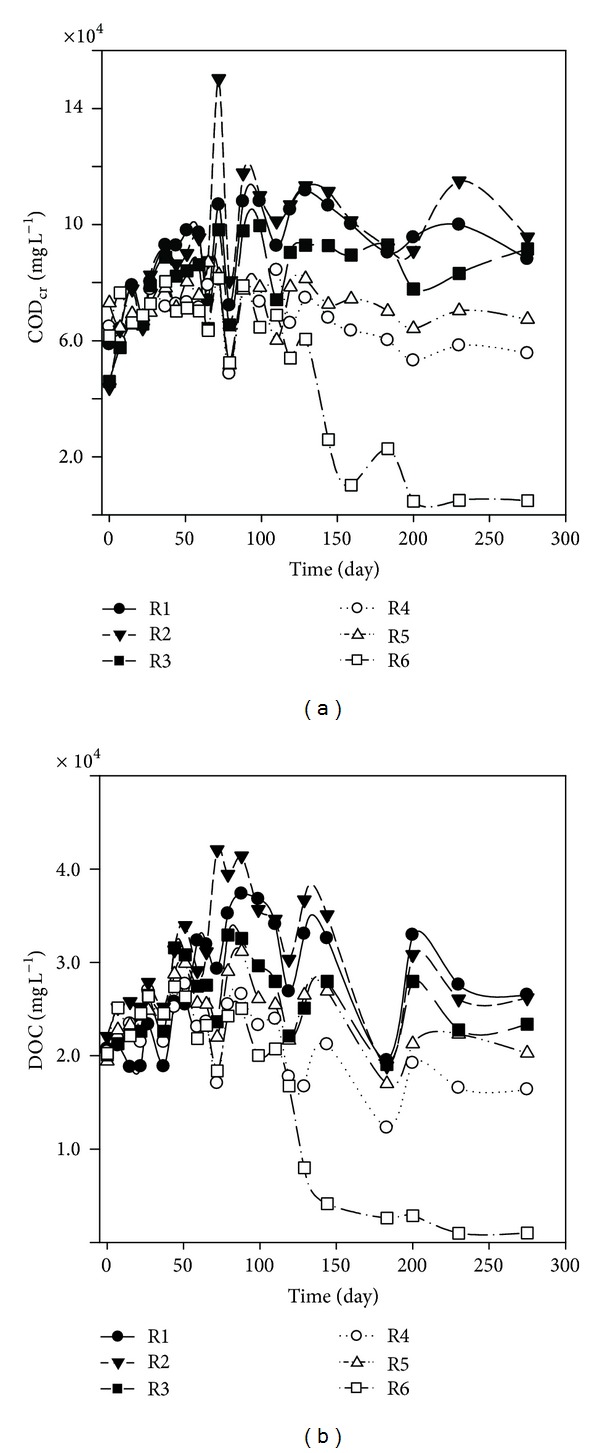
Time evolutions of COD_Cr_ (a) and DOC (b) in the leachate of the simulated landfill during operation.

As was shown in Figure 5(a), COD_Cr_ concentrations of the six simulated landfills increased rapidly, especially in the three simulated conventional landfills, due to the rapid release and hydrolysis of polymers, such as carbohydrates, fats, and proteins from the fresh refuse into the leachate. The changes of leachate COD_Cr_ concentration in the six simulated landfills were in accordance with the progression law of VFA and pH as the former elucidation in the study. The leachate COD_Cr_ concentrations of R1, R2, and R3 increased from 58700 mg L^−1^, 43800 mg L^−1^, and 46000 mg L^−1^ to 106800 mg L^−1^, 150200 mg L^−1^, and 98200 mg L^−1^ after 72-day operation, respectively. After two weeks, no significant change in the leachate COD_Cr_ concentrations was observed in the three simulated conventional landfills, and they were maintained within the range of 88100 mg L^−1^ to 111000 mg L^−1^ for R1, 91000 mg L^−1^ to 115000 mg L^−1^ for R2, and 74100 mg L^−1^ to 99600 mg L^−1^ for R3. The longer period for high level of COD_Cr_ in these simulated landfills might be attributed to the low populations and activity of methanogenic bacteria which only grow within a narrow pH range of 6.8 to 7.2 [22, 23]. The leachate COD_Cr_ concentrations of recirculated landfills were lower than CL, especially for R6 (the ratios of MSWI BA to fresh refuse was 1 : 5 (V : V)). After 72-day operation, the leachate COD_Cr_ concentrations of R4 and R5 increased from 64800 mg L^−1^ and 73000 mg L^−1^ to 81000 mg L^−1^ and 86900 mg L^−1^, respectively. From then on, the leachate COD_Cr_ concentrations of R4 and R5 decreased gradually and were maintained at 55600 mg L^−1^ and 67300 mg L^−1^ on day 275. The leachate COD_Cr_ concentration of R6 increased from 61700 mg L^−1^ to the maximum value of 81500 mg L^−1^ after 72 days operation and then kept within the range of 52400 mg L^−1^ to 78900 mg L^−1^ from day 99 to 129. From day 144, the leachate COD_Cr_ of R6 concentration decreased sharply and then was maintained approximately at 5000 mg L^−1^.

DOC is one of the main pollutants in MSW landfill leachate.  Figure 5(b) presented leachate DOC in the six simulated landfills over time. The changes in DOC in all landfills were basically in accordance with the progression law of COD_Cr_ as formerly elucidated. No significant difference was found in the initial leachate DOC concentrations of six simulated landfills, which were maintained around 20000 mg L^−1^. After 88-day operation, all the DOC concentrations reached peak values, which were varied with operational modes. The maximum values of the three simulated conventional landfills (R1, R2, and R3) kept within the range of 32500 mg L^−1^ to 41400 mg L^−1^, while it was 26500 mg L^−1^, 31200 mg L^−1^, and 25000 mg L^−1^, respectively, for R4, R5, and R6 on day 88. Afterwards, all the leachate DOC concentrations of six simulated landfills began to decrease, especially for R6, which decreased more rapidly than others. On day 129, the leachate DOC concentration of R6 was 7980 mg L^−1^, while the other five simulated landfills were present within the range of 16700 to 36700 mg L^−1^, which was two to four times higher than R6. Afterwards, the leachate DOC concentration of R6 continuously decreased and finally was maintained at about 1000 mg L^−1^. During the acid phase, leachate DOC content mainly consists of volatile fatty acids [24]. With the degradation of VFA, R6 passed from acid phase to methanogenic phase, and the DOC content correspondingly decreased and was maintained at a low level. However, the other five simulated landfills were still in the acid phase with high levels of leachate VFA and DOC concentrations.

### 3.4. Characteristics of Leachate Nitrogen

**Figure 6:**
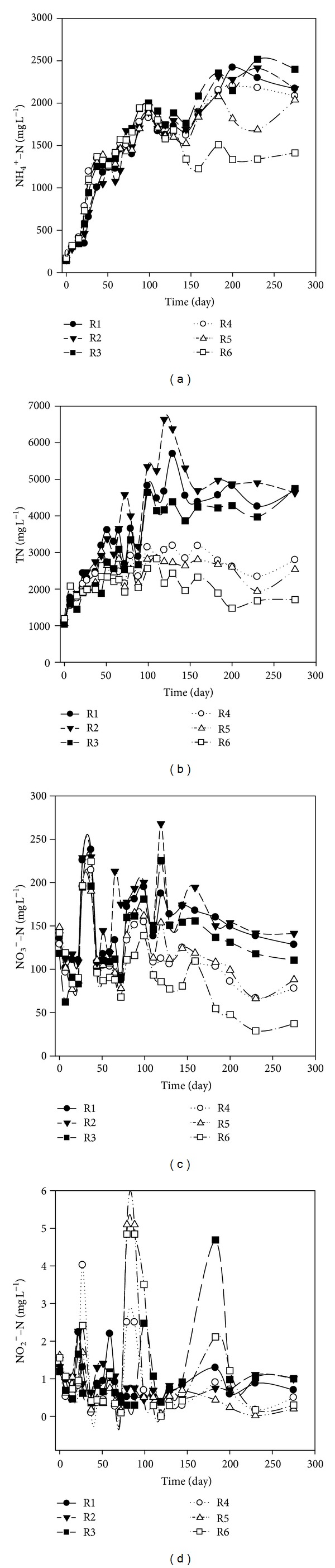
Time evolutions of NH_4_^+^-N (a), TN (b), NO_3_^−^-N (c), and NO_2_^−^-N (d) in the leachate of the simulated landfill during operation.

Ammonia was the major contributor to the total nitrogen in leachate as a result of the decomposition of nitrogenous matter, such as protein and amino acids. Apart from R6, the long-term high concentrations of ammonia were observed in the leachate in the other five simulated landfills during the whole operational process as reported previously [23, 25–27]. This phenomenon often occurs in anaerobic landfills. As was shown in Figure 6(a), the leachate NH_4_^+^-N concentrations in the six simulated landfills increased linearly and reached the peak value of 1820 mg L^−1^ to 2000 mg L^−1^ on day 99. Afterwards, in the simulated landfills of R1, R2, R3, R4, and R5, a V-shape pattern in the variation of leachate NH_4_^+^-N concentrations was observed, which firstly decreased to 1650 ± 80 mg L^−1^ from day 99 to day 144 and increased again and then was maintained around 2100 mg L^−1^. Our results are similar to those obtained by Bilgili et al. [28] and Huo et al. [19], suggesting that no mechanism of NH_4_^+^-N elimination occurred in anaerobic landfills [29]. On the contrary, an L-shape pattern was observed in leachate NH_4_^+^-N concentrations of R6 during the rest days. After reaching the peak value on day 99, leachate NH_4_^+^-N concentration of R6 decreased rapidly and then was maintained at approximately 1400 mg L^−1^. The different trends of NH_4_^+^-N concentrations between R6 and the other landfills might be attributed to the operational modes of the BA addition and leachate recirculation. As has been mentioned above, combining the BA addition with leachate recirculation could solve the ensiling problems and accelerate the process from acid phase to methanogenic-phase with a high pH value. The ammonium ion is mildly acidic, reacting with OH- to return to ammonia. Therefore, the degree to which ammonium ion changes to ammonia depends on the pH of the solution (see (1)). If the pH is low, the equilibrium shifts to the left: more ammonia molecules are converted into ammonium ions. On the contrary, if the pH is high, the equilibrium shifts to the right. As a result, the increase in leachate pH led to the decrease in NH_4_^+^-N concentration of R6:

(1) $$\begin{array}{c} {\text{N}{\text{H}}_{4}}^{+}+\text{O}{\text{H}}^{-}⟺\text{N}{\text{H}}_{3}↑+{\text{H}}_{2}\text{O}. \end{array}$$

The variations of TN concentrations were in accordance with those of the concentrations of NH_4_^+^-N in the simulated landfills of CL (Figure 6(b)). The leachate TN concentrations of R1, R2, and R3 increased linearly at the first 120 days and reached the maximum values of 5700 mg L^−1^, 6640 mg L^−1^, and 4390 mg L^−1^, respectively. Afterwards, the leachate TN concentrations of the three conventional landfills started to decrease and then were maintained about 4650 mg L^−1^. However, the leachate TN concentrations in the simulated landfills of RL presented different trends. After nearly 99-day operation, the leachate TN concentrations in the three simulated landfills increased gradually and reached the maximum values of 3140 mg L^−1^, 2830 mg L^−1^, and 2560 mg L^−1^, respectively, in R4, R5, and R6.

Afterwards, the leachate TN concentrations of R4 and R5 were maintained approximately at 2700 mg L^−1^ and 2500 mg L^−1^, respectively, with a little fluctuation on day 230. The leachate TN concentration of R6 decreased from the peak value of 2560 mg L^−1^ to 1700 mg L^−1^. The peak values and the final values of leachate TN concentration in the recirculated landfills both decreased as the ratio of BA addition was increased.

Two V-shape patterns in the NO_3_^−^-N concentrations were observed in the six simulated landfills at the beginning of 65 days, (Figure 6(c)). Afterwards, the leachate NO_3_^−^-N concentration decreased gradually and finally was maintained approximately at 130 mg L^−1^, 140 mg L^−1^, 110 mg L^−1^, 80 mg L^−1^, 90 mg L^−1^, and 40 mg L^−1^, respectively, in R1, R2, R3, R4, R5, and R6. During the whole operational process, the leachate NO_2_^−^-N concentration of the six simulated landfills was kept at 0-1.00 mg L^−1^ (Figure 6(d)). The highest concentration of NO_2_^−^-N was only 5.10 mg L^−1^ in the leachate of R6. Above all, addition of MSWI BA to landfill with the ratio of 1 : 5 and 1 : 10 (MSWI BA to fresh refuse, V : V) did not change the characteristics of leachate TN, which mainly consisted of NH_4_^+^-N in anaerobic landfills.

### 3.5. Implications

On the basis of leachate characteristics, addition of MSWI BA was beneficial to simulated landfill to reach a stable state. In view of the ratio of MSWI BA to fresh refuse, 1 : 5 (V : V) is better than 1 : 10 (V : V), since the former ratio would provide more sufficient acids neutralizing capacity to neutralize the volatile fatty acids in the leachate. Therefore, the ratio of MSWI BA to fresh refuse should be adjusted according to the change of organic composition in MSW. In addition to alkali metals, the BA also contains various types of heavy metals, which might be harmful to the microbes and further have a negative impact on the stabilization process of landfills. However, some researchers reported that heavy metals and trace metals in BA were too low to have inhibitory effects on anaerobic landfills. On the contrary, they have beneficial rather than detrimental effects on the landfills codisposing with MSWI BA and MSW [9–11]. In our experiment, the contents of copper (Cu) and zinc (Zn) in the BA were 314.6 ± 22.3 mg kg^−1^ and 1922.0 ± 33.0 mg kg^−1^, respectively, and higher than other heavy metals. No significant difference was found in the Cu concentrations in the leachate from the six simulated landfills. The codisposal with the ratio of MSWI BA to MSW of 1 : 10 (V : V) could increase the leachate Zn concentration, while the ratio of 1 : 5 (V : V) could decrease the releasing amount of Zn from the landfill due to the increase in pH value (data not shown here). Therefore, it seems that the heavy metal release from the waste via the leachate will not be influenced by the addition of MSWI BA. As was presented above, operational modes could also have significant impact on landfill stabilization, based on the leachate quality, especially in these codisposing landfills of MWSI BA and MSW. Without leachate recirculation, fewer amounts of alkali metals were released from MSWI BA for buffering the acid matters from landfilled refuse. Only the leachate acid from the upper side of the BA layer was neutralized. Therefore, the codisposal of MSWI and MSW could increase the contact opportunity between leachate acid and BA. However, Boni et al. [11] reported that disposal (mixed or layered) strategy did not have any significant effect on the leachate characteristics. In our study, the leachate quality of R6 was improved by the combination of the BA addition with leachate recirculation.

## 4. Conclusions

After 275-day operation, the results showed that both the ratio of MSWI BA to MSW and operational modes had significant impact on landfill stabilization. The ratio of MSWI BA to fresh refuse of 1 : 10 (V : V) was still not enough for high organic matter compositions of MSW (higher than 45.5%), while the ratio of MSWI BA to fresh refuse of 1 : 5 (V : V) could provide sufficient acid-neutralizing capacity for the landfill with a high content of organic waste. In addition, the leachate quality of landfills can be only improved by the operational modes with the BA addition and leachate recirculation.
